# Metformin Enhances B Cell Function and Antibody Responses of Elderly Individuals With Type-2 Diabetes Mellitus

**DOI:** 10.3389/fragi.2021.715981

**Published:** 2021-07-23

**Authors:** Daniela Frasca, Alain Diaz, Maria Romero, Bonnie B. Blomberg

**Affiliations:** ^1^ Department of Microbiology and Immunology, University of Miami Miller School of Medicine, Miami, FL, United States; ^2^ Sylvester Comprehensive Cancer Center, University of Miami Miller School of Medicine, Miami, FL, United States

**Keywords:** aging, Type-2 Diabetes Mellitus, B cells, inflammation, autoimmunity

## Abstract

Our previous work has shown that young and elderly patients with Type-2 Diabetes Mellitus (T2DM) treated with Metformin have optimal B cell function and serum antibodies specific for the seasonal influenza vaccine. In this paper, we have evaluated B cell function and the metabolic requirements of B cell antibody responses in elderly T2DM patients (E_T2DM_) taking or not Metformin, and compared to those of healthy elderly (E_H_) and healthy young (Y_H_) individuals. Results show that Metformin significantly increases *in vivo* B cell function, measured by influenza vaccine-specific serum antibodies, in E_T2DM_ patients to the levels observed in E_H_ and more importantly in Y_H_ individuals. Metformin also decreases the frequencies of pro-inflammatory B cell subsets, as well as intrinsic inflammation and metabolic requirements of peripheral B cells from E_T2DM_. This hyper-metabolic phenotype of B cells from E_T2DM_ is needed to support intrinsic inflammation, measured by the expression of transcripts for markers of the senescence-associated secretory phenotype (SASP), and the secretion of autoimmune antibodies. Importantly, B cell function in E_T2DM_ patients taking Metformin is not only increased as compared to that in E_T2DM_ patients not taking Metformin, but is comparable to B cell function measured in Y_H_ individuals. These results altogether strongly support the anti-aging effects of Metformin on humoral immunity.

## Introduction

Aging is associated with inflammaging known as the increased chronic low-grade serum inflammatory status (Franceschi et al., 2000), which is a significant risk factor for morbidity and mortality of older adults. Inflammaging is in fact involved in the pathogenesis of several debilitating chronic diseases. These include Type-2 Diabetes Mellitus (T2DM) (Prattichizzo et al., 2018). Inflammaging initiates and supports intrinsic inflammation in immune cells leading to reduced protective responses against infections and decreased humoral immunity to infections and vaccination (Bryl et al., 2001; Parish et al., 2009; Frasca et al., 2014). Inflammaging is also involved in metabolic dysfunction and in the development of insulin resistance (IR) (Hotamisligil, 2017).

T2DM is one of the most prevalent chronic inflammatory disease of older adults (https://www.cdc.gov/diabetes/data/statistics-report/newly-diagnosed-diabetes.html). It is a metabolic disease not associated with autoimmunity, but often characterized by obesity, hypertension, dyslipidemia, accelerated atherosclerosis, and increased mortality (Alberti and Zimmet, 1998). Its development has been related to inflammaging, in particular to the acquisition of the senescence-associated secretory phenotype (SASP) and related oxidative stress and endoplasmic reticulum stress (Prattichizzo et al., 2016), two processes also associated with physiological aging (Muriach et al., 2014; Bonomini et al., 2015). Life expectancy of T2DM patients has been reported to be about 6 years shorter than that of age-matched healthy controls, mainly due to the increased risk for progressive disability due to T2DM-associated chronic diseases and increased age (Huang et al., 2014). In contrast, catastrophic disability has been attributed to illness events associated with infections, including respiratory tract infections with influenza and associated complications (pneumonia, ischemic heart disease, congestive heart failure, and stroke) (Guralnik et al., 2001), and pulmonary tuberculosis (Prada-Medina et al., 2017), as well as with urinary tract infections (Nitzan et al., 2015).

Immune responses are dysfunctional in both young and elderly T2DM patients, leading to increased susceptibility to get infections and reduced responses to vaccination as compared to healthy age-matched controls, and these defects are exacerbated in older patients, due to their increased inflammatory condition (Pozzilli et al., 1986; Smith and Poland, 2000; Muller et al., 2005; McElhaney et al., 2015). Hyperglycemia in T2DM is considered to be a cause of dysfunctional immune responses, as it is associated with several pro-inflammatory and metabolic pathways, including the advanced glycated end products pathway (Singh et al., 2014) and the reactive oxygen intermediate pathway (Yan, 2014), which lead to increased intrinsic inflammation in immune cells and decreased function.

Metformin (dimethyl biguanide) is a synthetic product of guanidine, initially isolated from the extracts of a plant with anti-diabetic effects (Galega officinalis). Since its discovery more than 50 years ago, Metformin is the first-line medication for T2DM patients. Metformin is currently the only hypoglycemic and anti-inflammatory drug also influencing cellular processes associated with the development of chronic conditions of old age (inflammaging, oxidative damage, increased glycation of proteins, cell senescence, apoptosis). Recent findings in humans have shown that Metformin has anti-proliferative (Sabry et al., 2019), anti-fibrotic (Wang et al., 2020) and anti-oxidant (Esteghamati et al., 2013) effects, suggesting its potential use as an anti-aging molecule. Although not fully understood, the pleiotropic effects of Metformin are thought to be mediated primarily through the regulation of AMPK [5′-adenosine monophosphate (AMP)-activated protein kinase] and mTOR (mammalian target of rapamycin). AMPK activity is decreased in the liver, muscle, and adipose tissue of individuals with IR (Ruderman and Prentki, 2004).

We have previously shown that young and elderly T2DM patients treated with Metformin have optimal B cell function and humoral immunity to the seasonal influenza vaccine (Frasca et al., 2013a). In this paper, we have further evaluated B cell function and the metabolic requirements of B cell antibody responses in the following individuals: elderly T2DM patients (E_T2DM_) taking or not Metformin, healthy elderly (E_H_) and healthy young (Y_H_) individuals. Results show that Metformin significantly increases *in vivo* influenza vaccine-specific serum antibodies in E_T2DM_ patients. Metformin also decreases intrinsic inflammation in B cells from E_T2DM_ and their hyper-metabolic phenotype needed to support the expression of markers of the senescence-associated secretory phenotype (SASP), as well as the secretion of pathogenic autoimmune antibodies.

## Materials and Methods

### Subjects

For this study, we recruited at the University of Miami Miller School of Medicine elderly patients with T2DM (E_T2DM_) taking Metformin (*n* = 14, age 74 ± 2) or not (*n* = 4, age 72 ± 2). Controls were healthy elderly (E_H_, *n* = 9, age 76 ± 3), and young (Y_H_, *n* = 14, age 35 ± 1) individuals. The limited number of T2DM patients not taking Metformin is due to the mandatory rule of the University of Miami that patients diagnosed with T2DM must start treatment immediately after they have been diagnosed. After the establishment of this rule, it has been impossible to recruit treatment naïve T2DM patients. Both T2DM patients and healthy participants were screened for diseases known to alter the immune response or for consumption of medications that could alter the immune response. Subjects with autoimmune diseases, congestive heart failure, cardiovascular disease, chronic renal failure, malignancies, renal or hepatic diseases, infectious disease, trauma or surgery, pregnancy, or documented current substance and/or alcohol abuse were excluded. Dose of Metformin taken by E_T2DM_ patients was 500 mg twice/day. E_T2DM_ patients were on Metformin for at least 2 years before their recruitment.

All participants signed an informed consent. The study was reviewed and approved by our Institutional Review Board (IRB, protocols #20070481 and #20160542), which reviews all human research conducted under the auspices of the University of Miami.

### Influenza Vaccination

Study participants were recruited during the 2011–2012, 2012–2013, and 2013–2014 influenza vaccine seasons. The 2011–2012 vaccine contained A/California/7/2009 (H1N1), A/Perth/16/2009 (H3N2), and B/Brisbane/60/2008. The 2012–2013 vaccine contained A/California/7/2009 (H1N1), A/Victoria/361/2011 (H3N2), and B/Wisconsin/1/2010-like (Yamagata lineage). The 2013–2014 vaccine contained A/California/7/2009 (H1N1), A(H3N2) virus antigenically like the cell-propagated prototype virus A/Victoria/361/2011 and B/Massachusetts/2/2012.

All participants at the time of enrollment were influenza-free, without symptoms associated with respiratory infections and did not contract flu-like symptoms within a 6-months follow-up period.

Blood samples were collected immediately before and 4 weeks after vaccination.

### Hemagglutination Inhibition Assay

We evaluated H1N1-specific titers after vaccination, as we have previously described (Frasca et al., 2010; Frasca et al., 2012a; Frasca et al., 2013a; Frasca et al., 2013b), as the same H1N1 was repeated in the three consecutive seasons. The Hemagglutination Inhibition (HAI) assay is useful for the measurement of antibody titers in serum and is the most established correlate with vaccine protectiveness (Murasko et al., 2002; Skowronski et al., 2008).

### PBMC Collection

Blood was drawn in Vacutainer CPT tubes (BD 362761). PBMC were isolated and cryopreserved. PBMC (1 × 10^6^/ml) were thawed and cultured in complete medium (c-RPMI, RPMI 1640, supplemented with 10% FCS, 10 μg/ml Pen-Strep, 1mM Sodium Pyruvate, and 2 × 10^−5^ M 2-ME and 2 mM l-glutamine). After thawing the PBMC, viability is checked. We discard samples if viability is <75%, as evaluated by trypan blue counting.

### B Cell Isolation and *In vitro* Stimulation

B cells were isolated from thawed PBMC by magnetic sorting using CD19 Microbeads (Miltenyi) and manufacturer’s instructions. Cell preparations were typically >95% pure. For autoimmune antibody production, B cells were stimulated for 10 days in c-RPMI with CpG (invivoGen ODN 2006, 10 μg/ml), then supernatants were collected and IgG antibodies measured by ELISA.

### Flow Cytometry

After thawing, PBMC (2 × 10^6^/ml) were stained for 20 min at room temperature with the following antibodies: anti-CD19 (BD 555415), anti-CD27 (BD 555441), and anti-IgD (BD 555778) to measure naive (IgD + CD27−), IgM memory (IgD + CD27+), switched memory (swIg, IgD-CD27+), and Double Negative (DN, IgD-CD27−) B cells.

To measure glucose uptake, we used the glucose fluorescent analog [2-(N-(7-Nitrobenz-2-oxa-1,3-diazol-4-yl)Amino)-2-Deoxyglucose] (2-NBDG, Thermo Fisher N13195). Briefly, PBMCs (10^6^/ml) were left unstimulated and then 2-NBDG was added at a final concentration of 50 μM for 30 min.

Up to 10^5^ events in the B cell gate were acquired on an LSR-Fortessa (BD) and analyzed using FlowJo 10.5.3 software. In every experiment we included single color controls for compensation purposes as well as isotype antibodies to set up the gates.

### mRNA Extraction and Quantitative PCR

To evaluate mRNA expression of the glucose transporter Glut1 and of SASP markers, the mRNA was extracted from B cells, using µMACS magnetic beads (Miltenyi) and manufacturer’s instructions. The mRNA was eluted into 75 µL of pre-heated (65°C) elution buffer, and stored at −80°C until use. Reverse Transcriptase (RT) reactions were performed in a Mastercycler Eppendorf Thermocycler to obtain cDNA. Briefly, 10 µL of mRNA +10 µL of RT-mix were used for cDNA synthesis. Conditions were: 40 min at 42°C and 5 min at 65°C.

Five µL of cDNA were used for qPCR. Reactions were conducted in MicroAmp 96-well plates and run in the ABI 7300 machine. Calculations were made with ABI software. For calculations, we determined the cycle number at which transcripts reached a significant threshold (Ct) for both target genes and for GAPDH (control). The difference in Ct values between GAPDH and each of the target genes was calculated as ΔCt. Then the relative amount of the target gene was expressed as 2^−ΔCt^ and indicated as qPCR values. Reagents and Taqman primers, all from Life Technologies, were the following: GAPDH, Hs99999905_m1; TNF, Hs01113624_g1; IL-6, Hs00985639_m1; p16^INK4^ (CDKN2A), Hs00923894_m1; Glut1 (SLC2A1), Hs00892681_m1.

### Metabolic Measurements

We used a Mitostress test to evaluate oxygen consumption rates (OCR), a measure of oxidative phosphorylation (OXPHOS), and extracellular acidification rates (ECAR), a measure of anaerobic glycolysis. The Mitostress test was conducted in a Seahorse XFp extracellular flux analyzer (Agilent). Briefly, we seeded B cells from E_T2DM_ patients taking Metformin or not, as well as from E_H_ and Y_H_ individuals, in a CellTAK (BD Biosciences)-coated plate, at the concentration of 2.5 × 10^5^/well in XF DMEM medium supplemented with glutamine, glucose, and pyruvate (200 μL of each reagent in 20 ml of medium). Maximal respiratory capacity was measured by the addition of the following compounds: Oligomycin (1 μM) to block ATP production, and then FCCP (fluoro-carbonyl cyanide phenylhydrazone, 5 μM), an uncoupling agent, to dissipate proton gradients and allow electron transport and oxygen consumption to operate at maximal rate. Maximal respiration was then suppressed by the addition of Rotenone/Antimycin (1 μM), showing that respiration is mitochondrial.

### ELISA to Measure Autoimmune Antibodies in Culture Supernatants

For dsDNA-specific IgG antibodies we used the Signosis EA-5002 kit, following manufacturer’s instructions.

### Statistical Analyses

To examine differences between four groups, two-way ANOVA was used. Group-wise differences were analyzed afterwards with Bonferroni’s multiple comparisons test, with *p* < 0.05 set as criterion for significance. To examine differences between two groups, unpaired Student’s t tests (two-tailed) were used. To examine relationships between variables, bivariate Pearson’s correlation analyses were performed. GraphPad Prism version 8.4.3 software was used to construct all graphs.

## Results and Discussion

### Metformin Enhances the Serum H1N1-Specific Antibody Response of E_T2DM_ Patients to the Levels Observed in E_H_ and More Importantly in Y_H_ Individuals

**FIGURE 1:**
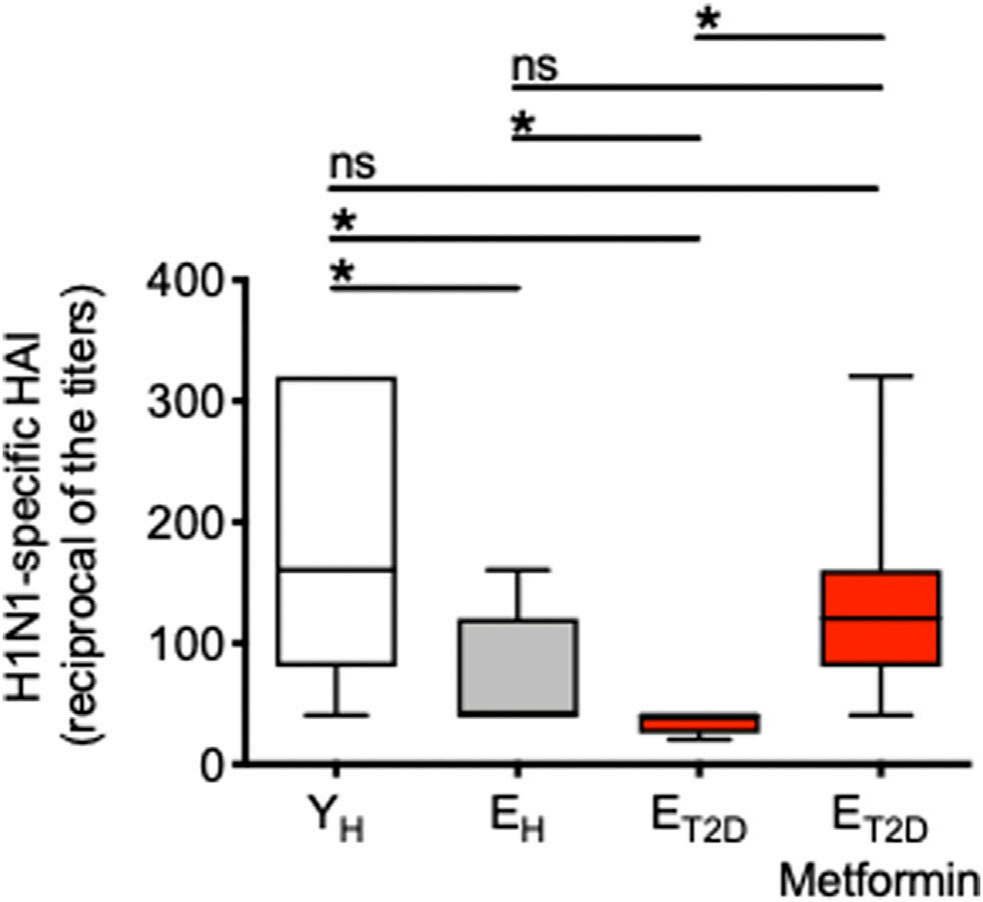
Metformin significantly enhances the serum H1N1-specific antibody response of E_T2DM_ patients to the levels observed in E_H_ and more importantly in Y_H_ individuals. Serum samples were collected from the 4 groups of individuals and evaluated by H1N1-specific HAI. Results show the reciprocal of the titers 4 weeks after vaccination. Mean comparisons between groups were performed by one-way ANOVA. *p < 0.05.

We measured the serum H1N1-specific antibody response by HAI in E_T2DM_ patients taking Metformin or not, as well as in E_H_ and Y_H_ individuals. Results in Figure 1 show that the response is significantly decreased in E_H_ as compared to Y_H_ individuals. The response is decreased even more in E_T2DM_ patients as compared to E_H_ individuals. Metformin significantly enhances the response of E_T2DM_ patients to the levels observed in E_H_ and more importantly in Y_H_ individuals.

These results clearly indicate that T2DM superimposed on aging further decreases influenza vaccine responses in elderly individuals, and suggest the need to treat E_T2DM_ patients with Metformin as soon as they are diagnosed in order to improve their response and protect them from the risk of infection.

### Metformin Decreases the Frequencies of Pro-Inflammatory B Cell Subsets in the Blood of E_T2DM_ Patients

**FIGURE 2:**
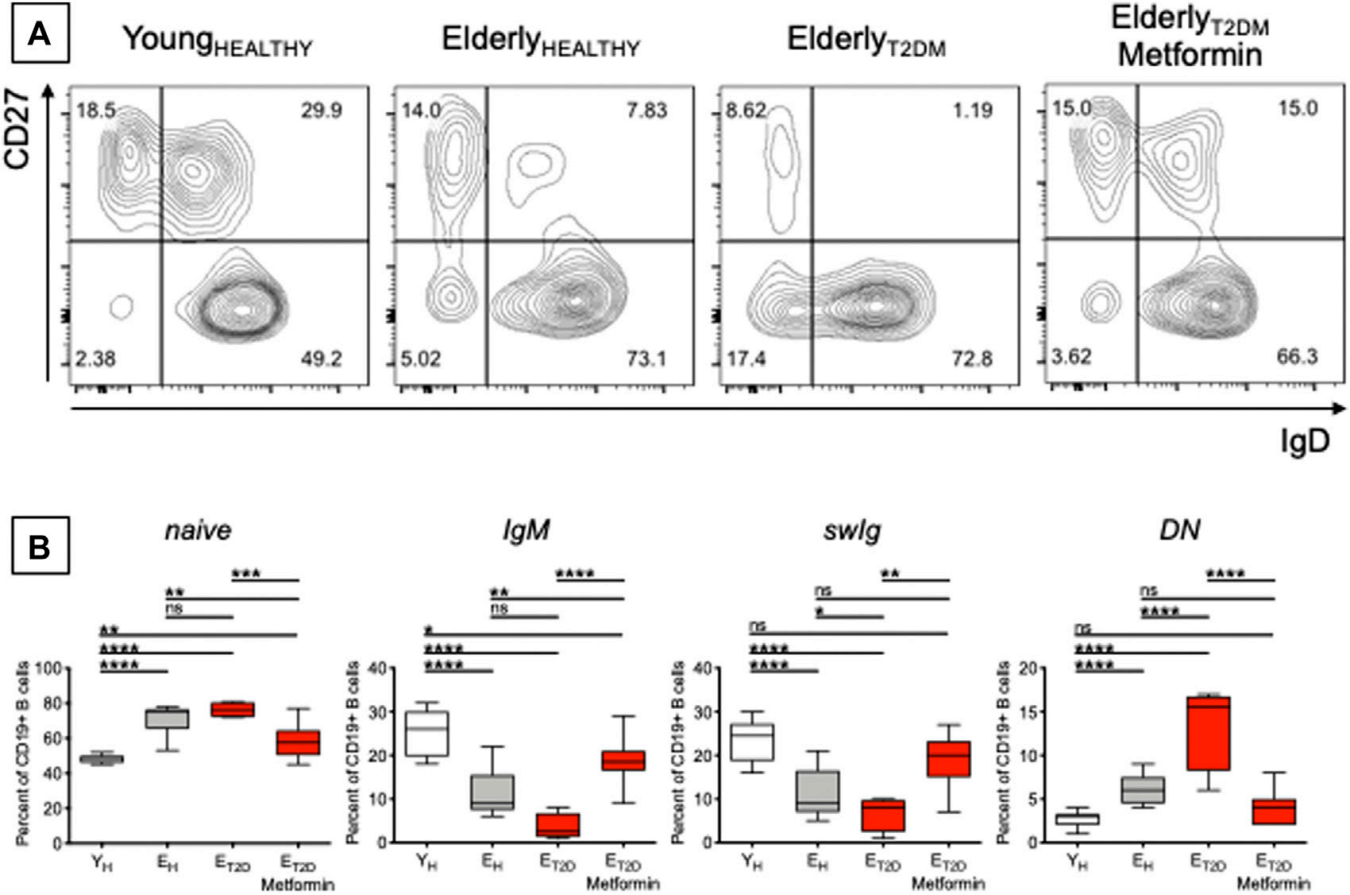
Metformin decreases the frequencies of pro-inflammatory B cell subsets in the blood of E_T2DM_ patients. **(A)** Gating strategies and a representative contour plot from one individual/group. In this representative plot, naïve (IgD + CD27−) are in the lower right quadrant, IgG memory (IgD + CD27+) are in the upper right quadrant, swIg (IgD-CD27+) are in the upper left quadrant and DN (IgD-CD27-) are in the lower left quadrant. **(B)** Frequencies of the 4 B cell subsets in the 4 groups of individuals. Mean comparisons between groups were performed by one-way ANOVA. *p < 0.05, **p < 0.01, ***p < 0.001, ****p < 0.0001.

The composition of the peripheral B cell pool influences the individual’s response to the influenza vaccine, and it has been shown that serum vaccine-specific antibody responses are reduced in E_H_ as compared to Y_H_ individuals, in part due to decreased generation of the subset of swIg B cells (Amanna et al., 2007; Sasaki et al., 2008; Frasca et al., 2010; Frasca et al., 2012b; Frasca et al., 2013a) and of long-lived plasma cells residing in the bone marrow (Sasaki et al., 2011; Pritz et al., 2015). We therefore measured the frequencies of the major B cell subsets present in blood of E_T2DM_ patients taking Metformin or not, and in E_H_ and Y_H_ individuals. Previously published results have shown that aging increases the frequencies of naïve B cells (Chong et al., 2005; Shi et al., 2005; Frasca et al., 2016; Frasca et al., 2017a; Frasca et al., 2017b), although some studies have also reported the opposite result, a decrease in naïve B cell frequencies with age (Colonna-Romano et al., 2003; Buffa et al., 2013), likely due to differences among cohorts evaluated. Aging also increases the frequencies of DN B cells (Frasca et al., 2008; Colonna-Romano et al., 2009; Frasca et al., 2016; Frasca et al., 2017a; Frasca et al., 2017b) and concomitantly decreases the frequencies of IgM (Shi et al., 2005) and swIg (Shi et al., 2005; Frasca et al., 2008; Frasca et al., 2016; Frasca et al., 2017a; Frasca et al., 2017b) memory B cells. Results in Figure 2 confirm these previously published results and also show that T2DM induces further changes, exacerbating the effects of aging. Briefly, Figure 2A shows a representative experiment in which B cells isolated from the blood of the four groups of participants were stained with anti-CD19, anti-CD27, and anti-IgD antibodies to evaluate the frequencies of the major B cell subsets. Results from all the particpants are shown in Figure 2B. The composition of the peripheral B cell pool of E_T2DM_ patients taking Metformin is comparable to that from Y_H_ individuals, and especially for the subsets of swIg and DN B cells.

These are to our knowledge the first results showing decreased frequencies of DN B cells in the blood of E_T2DM_ patients taking Metformin as compared to those not taking Metformin. DN B cells are the most pro-inflammatory B cell subset (Frasca et al., 2016; Frasca et al., 2019). These cells increase in the blood of individuals with inflammatory conditions and diseases, such as physiological aging (Colonna-Romano et al., 2009; Frasca et al., 2017a; Nevalainen et al., 2019), obesity (Frasca et al., 2016; Frasca et al., 2019; Frasca et al., 2021a), autoimmune diseases (Wehr et al., 2004; Saadoun et al., 2013; Martorana et al., 2014; Adlowitz et al., 2015; Claes et al., 2016; Wang et al., 2018; Golinski et al., 2020), chronic (Moir et al., 2008; Illingworth et al., 2013; Chang et al., 2017), and acute (Woodruff et al., 2020) infectious diseases. DN B cells are characterized by high RNA expression of several pro-inflammatory markers, such as cytokines (TNF, IL-6), chemokines (IL-8), micro-RNAs, miRs (miR-155, miR-16, miR-181a), and cell cycle inhibitors and markers of proliferation arrest (p16^INK4^, p21^CIP1/WAF1^, and p53) (Frasca et al., 2017a; Frasca et al., 2021b), thus contributing significantly to inflammaging. Based on the results in Figure 2, we can hypothesize that Metformin may be a valid anti-inflammatory treatment also for the above inflammatory conditions and diseases in which DN B cells play a crucial role in the maintenance of chronic inflammation.

### Metformin Decreases mRNA Expression of SASP Markers in Ex Vivo-Isolated B Cells from E_T2DM_ Patients

**FIGURE 3:**
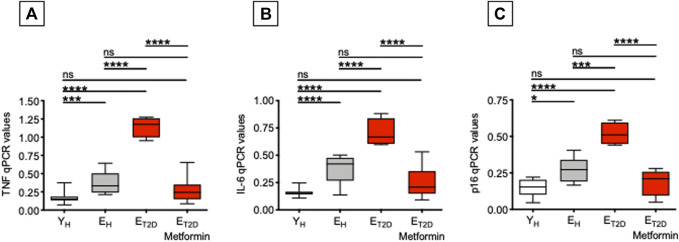
Metformin decreases mRNA expression of SASP markers in ex vivo-isolated B cells from E_T2DM_ patients. B cells were resuspended in TRIzol, then the RNA was extracted and the expression of SASP markers detected by qPCR to evaluate expression of RNA for the pro-inflammatory cytokines TNF-α **(A)** and IL-6 **(B)**, and for the cell cycle regulator p16^INK4^ **(C)**. qPCR values are measures of RNA expression of target genes, relative to the housekeeping gene GAPDH, calculated as 2^−ΔCts^. Mean comparisons between groups were performed by one-way ANOVA. *p < 0.05, ***p < 0.001, ****p < 0.0001.

We have previously shown that serum antibodies generated in response to the influenza vaccine are negatively associated with TNF-α mRNA and protein expression in ex vivo-isolated B cells from both young and elderly individuals (Frasca et al., 2014; Frasca et al., 2016), as well as with the frequencies of DN B cells (Frasca et al., 2014; Frasca et al., 2017a). Therefore, we measured in B cells from E_T2DM_ patients taking Metformin or not, as well as in B cells from E_H_ and Y_H_ individuals, mRNA expression of the pro-inflammatory cytokines TNF-α and IL-6, and of the cell cycle inhibitor p16^INK4^, all markers of the SASP (Coppé et al., 2010). Results in Figure 3 show that Metformin significantly decreases mRNA expression of TNF-α and IL-6 as well as of p16^INK4^ in B cells from E_T2DM_ patients to levels comparable to those observed in B cells from E_H_ and Y_H_ individuals. This result is likely reflecting the effect of Metformin in reducing DN B cell frequencies. In the 4 groups of participants, TNF-α, IL-6, and p16^INK4^ levels were found negatively associated with the serum H1N1-specific antibody response (Pearson’s *r* = −0.61, *p*=0.012 for TNF-α; *r* = −0.72, *p* = 0.002 for IL6, *r* = −0.62, *p* = 0.010 for p16^INK4^).

These results demonstrate that Metformin is an effective treatment for T2DM-associated B cell intrinsic inflammation, known to negatively affect influenza vaccine responses (Frasca et al., 2014). Although not directly demonstrated in the present study, we hypothesize that Metformin may prevent negative effects of TNF-α on B cells, possibly through decreased TNF-α signaling and reduced NF-kB activation, as it has already been shown in other cell types (Isoda et al., 2006; Li et al., 2009; Gu et al., 2014; Sekino et al., 2018; Kanigur Sultuybek et al., 2019).

### Metformin Decreases Glucose Uptake in Ex Vivo-Isolated B Cells From Elderly T2DM Patients

Our recently published results in mice have shown that the higher expression of SASP markers in B cells isolated from the spleens of old mice is associated with a hyper-metabolic profile needed to support their pro-inflammatory phenotype (Frasca et al., 2021c). Splenic B cells from old mice, as compared to those from young mice, are characterized by higher glucose uptake and higher mRNA expression of key metabolic enzymes involved in anaerobic glycolysis and OXPHOS, such as lactate dehydrogenase that converts pyruvate into lactate and represents a measure of anerobic glycolysis and pyruvate dehydrogenase that converts pyruvate into acetyl-CoA and represents a measure of OXPHOS and mitochondrial function.

**FIGURE 4:**
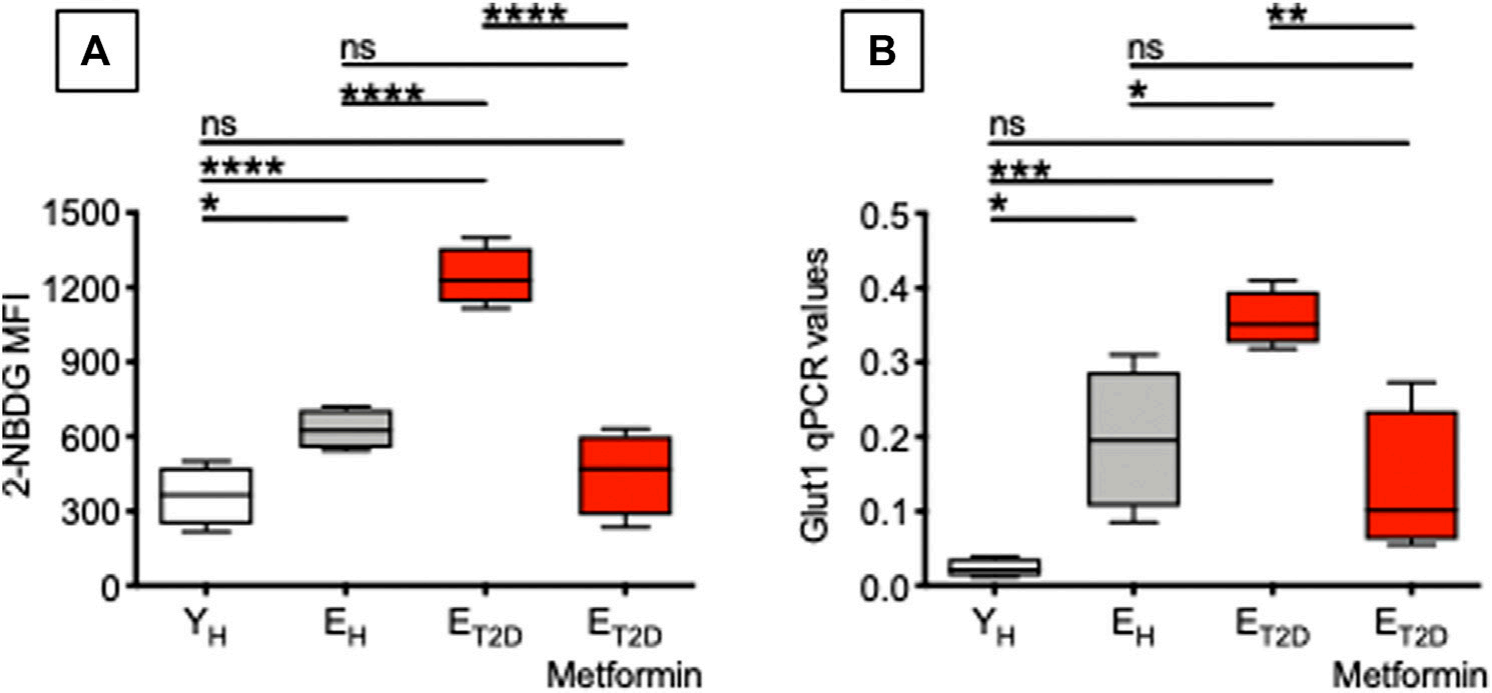
Metformin decreases glucose uptake in ex vivo-isolated B cells from E_T2DM_ patients. Glucose uptake was measured by flow cytometry and the glucose fluorescent analog 2-NBDG. **(A)** MFI (mean fluorescence intensity) of 2-NBDG staining in B cells from the 4 groups of individuals. **(B)** qPCR values of Glut1 mRNA expression, relative to GAPDH, calculated as 2^−ΔCts^ in the same B cells in **(A)**. Mean comparisons between groups were performed by one-way ANOVA. *p < 0.05, **p < 0.01, ***p < 0.001, ****p < 0.0001.

Therefore, we measured glucose uptake in ex vivo-isolated B cells from E_T2DM_ patients taking Metformin or not (four patients/group), as well as in B cells from E_H_ and Y_H_ individuals (four individuals/group). We used flow cytometry and the 2-NBDG glucose fluorescent analog. Results in Figure 4 show that Metformin decreases glucose uptake in B cells from E_T2DM_ patients to levels observed in B cells from E_H_ and Y_H_ individuals (A). Metformin also decreases the mRNA expression of the glucose transporter Glut1 (B).

**FIGURE 5:**
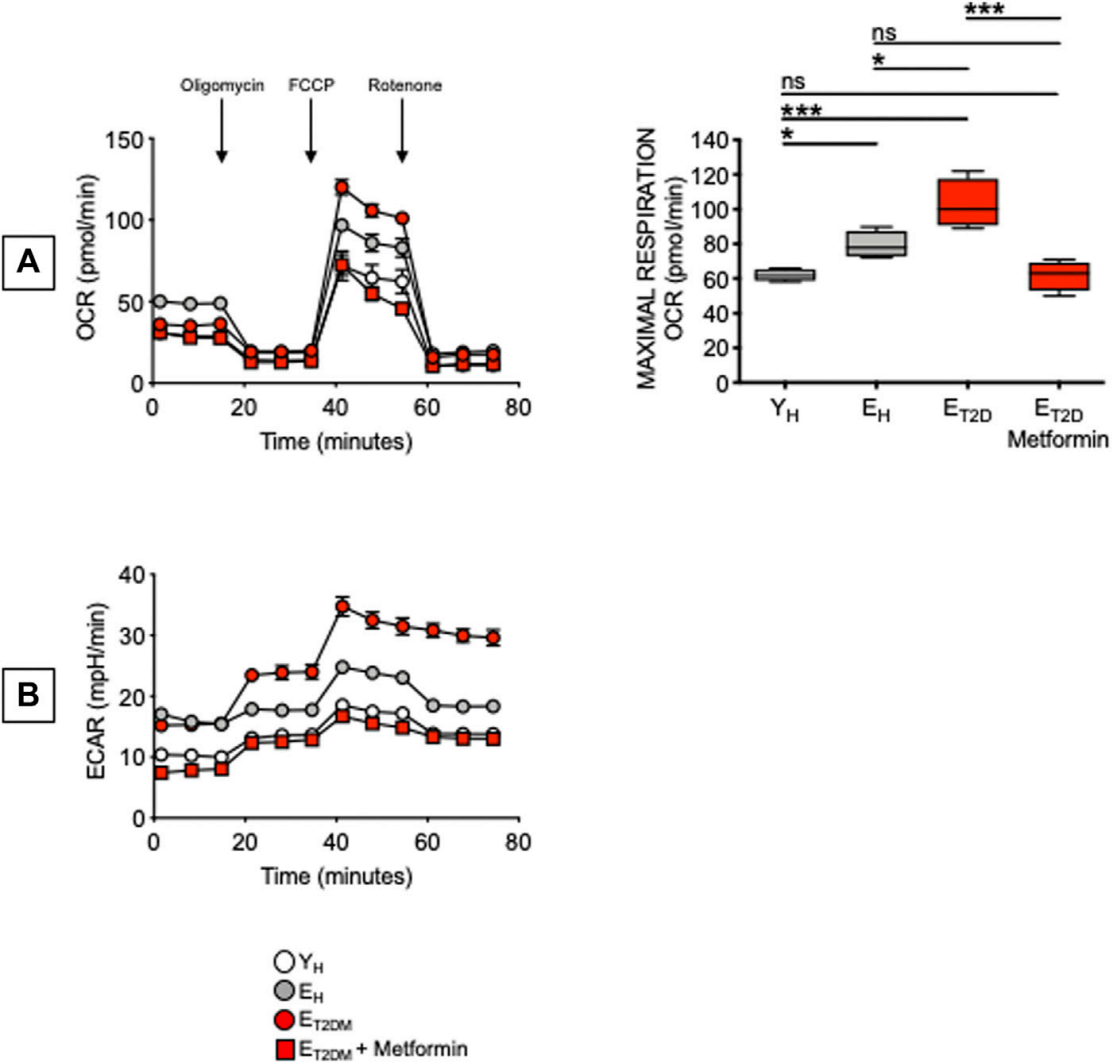
Metformin decreases OCR and ECAR in ex vivo-isolated B cells from E_T2DM_ patients. B cells were seeded into the wells of an extracellular flux analyzer at the concentration of 2 × 10^5^/well in triplicate and run in a mitostress test. **(A)** OCR results (left) and maximal respiration results (right) are shown. Mean comparisons between groups were performed by one-way ANOVA. *p < 0.05, ***p < 0.001. **(B)** ECAR results.

Glucose uptake is associated with a higher metabolic phenotype. Therefore, we evaluated the metabolic profile of B cells from the same patients and healthy controls in Figure 4. We performed a mitostress test comparing B cells from E_T2DM_ patients taking Metformin or not, as well as from E_H_ and Y_H_ individuals (4 individuals/group). Magnetic beads-sorted B cells from the 4 groups of participants were seeded into the wells of an extracellular flux analyzer to evaluate real-time changes in OCR and ECAR. Seahorse technology gives the possibility to perform several measures of mitochondrial function, including maximal respiration and spare respiratory capacity with a relatively high throughput. Results in Figure 5 show, as expected, higher OCR (A, left) and ECAR (B) in B cells from E_T2DM_ patients not taking Metformin as compared to those from E_T2DM_ patients on Metformin, confirming the higher inflammatory profile of B cells from E_T2DM_ patients not taking Metformin. Moreover, Metformin decreases OCR and ECAR in B cells from E_T2DM_ patients to levels observed in B cells from Y_H_ individuals, and lower as compared to B cells from E_H_ individuals. Major differences in OCR among the 4 groups were observed in maximal respiration (A, right).

These results are the first to our knowledge to show that Metformin decreases glucose uptake, OCR and ECAR in B cells from E_T2DM_ patients. In the 4 groups of participants, 2-NBDG MFI measures and maximal respiration were also found negatively associated with the serum H1N1-specific antibody response (Pearson’s *r* = −0.61, *p* = 0.015, and *r* = −0.59, *p* = 0.015, respectively). It was recently shown that Metformin controls intrinsic inflammation by decreasing OCR as well as OCR:ECAR ratio in Th17 cells from E_T2DM_ patients (Bharath et al., 2020). Importantly, in this T cell study Metformin was effective in shifting measures of mitochondrial function and intrinsic inflammation to values indistinguishable from those observed in cells from young individuals. These findings together with ours in Figure 5 highlight the relationship between metabolism and intrinsic inflammation and provide examples of how metabolic pathways can represent potential novel therapeutic targets, supporting the importance of clinical trials to improve health span with Metformin.

### Metformin Decreases Autoimmune Antibody Secretion in CpG-Stimulated B Cells From Elderly Type-2 Diabetes Mellitus Patients

**FIGURE 6:**
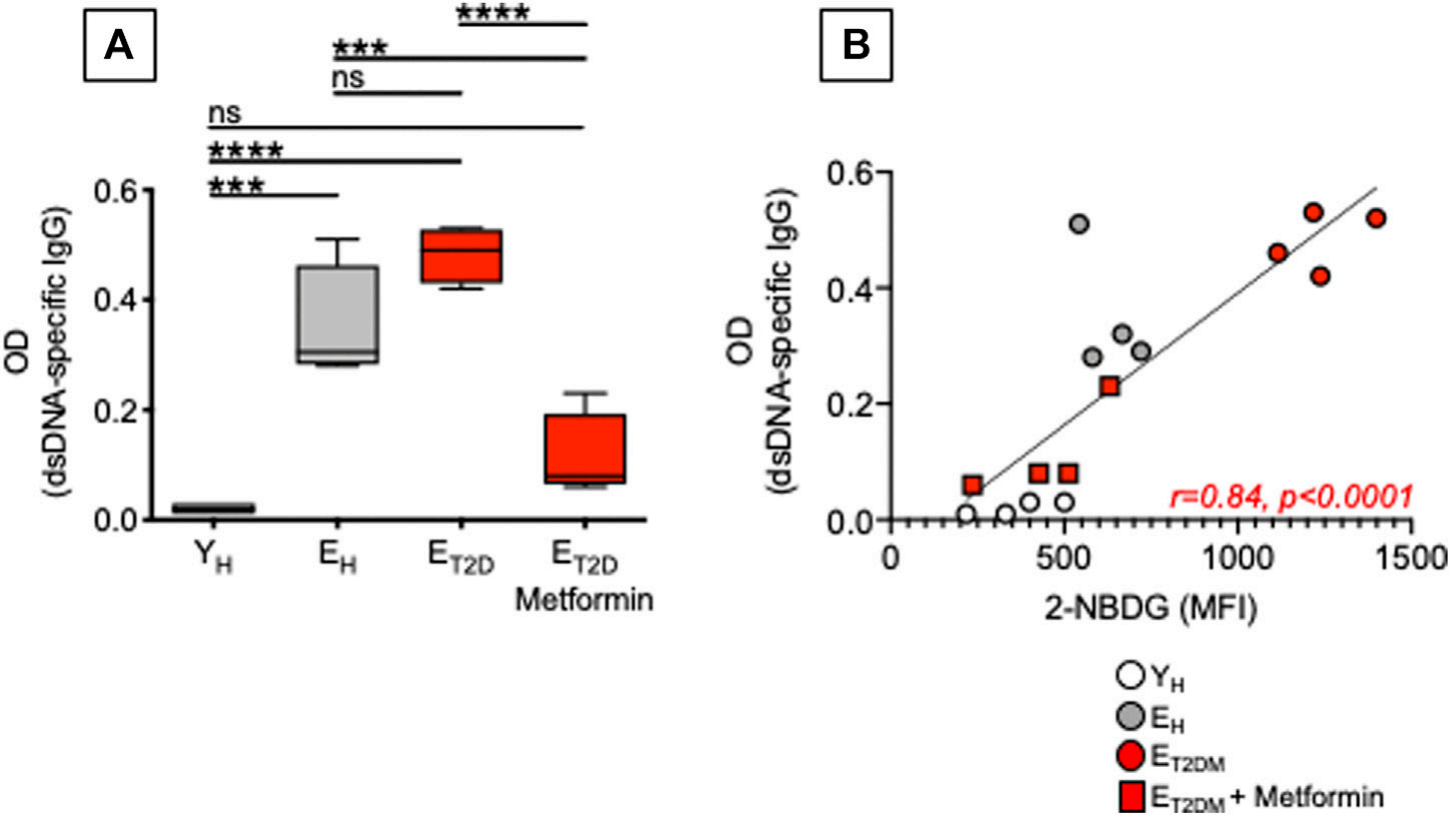
Metformin decreases autoimmune antibody secretion in CpG-stimulated B cells from E_T2DM_ patients. **(A)** Results show OD values at 1:200 sample dilution. **(B)** Correlations of anti-dsDNA IgG (OD values) with 2-NBDG MFI. Pearson’s regression coefficients and *p* values are indicated at the bottom of the figure.

Our previously published work has shown that a higher metabolic phenotype is associated with the secretion of IgG antibodies with autoimmune specificities in both mice (Frasca et al., 2021c; Caro-Maldonado et al., 2014) and humans (Frasca et al., 2019). Here, we measured the secretion of anti-dsDNA IgG antibodies in culture supernatants of CpG-stimulated B cells isolated from E_T2DM_ patients taking Metformin or not, as well as of B cells isolated from E_H_ and Y_H_ individuals (same patients and healthy controls in Figure 4). Although T2DM is not a typical autoimmune disease, autoimmune antibodies with different specificities can be detected in the majority of these patients (de Candia et al., 2019). Results in Figure 6 show that Metformin decreases autoimmune IgG secretion in B cells from E_T2DM_ patients as compared to B cells from E_H_ and Y_H_ individuals (A). As expected, glucose uptake and autoimmune IgG antibodies were positively associated (B).

Although Metformin has been shown to be effective in reducing inflammation in various models of autoimmune diseases such as systemic lupus erythematosus (Yin et al., 2015), colitis (Lee et al., 2015), and experimental autoimmune arthritis (Son et al., 2014), its effects on autoimmune B cells have not been evaluated yet. Our results herein are particularly important as it is known that upregulation of glucose uptake and glycolysis are essential for B cell-mediated generation of pathogenic autoimmune Th cells, and immunotherapy with anti-CD20 antibodies, that specifically depletes B cells, completely abrogates glucose uptake in Th cells and suppresses disease progression in patients with autoimmune diseases (Zeng et al., 2020).

## Conclusion

Results herein show that Metformin significantly increases the antibody response to the influenza vaccine in E_T2DM_ patients to the levels observed in E_H_, and more importantly in Y_H_ individuals, likely by decreasing B cell intrinsic inflammation that is negatively associated with protective responses to infections and vaccination. Our results also show that Metformin induces changes in the metabolic phenotype of B cells, reducing their hyper-metabolic status that is supporting age-associated B cell intrinsic inflammation, the expression of SASP markers and the secretion of pathogenic autoimmune antibodies. Although many studies have already outlined the importance of Metformin in the regulation of metabolic requirements of T cells, studies on the effects of Metforming on B cell metabolism are lacking. Our results therefore add important findings indicating that Metformin represents an effective therapeutic option to rewire metabolic pathways also in B cells, leading to improved humoral immunity and reduced inflammation and autoimmunity.

## Data Availability

The raw data supporting the conclusion of this article will be made available by the authors, without undue reservation.
